# Total Hip Arthroplasty Concomitant with Patellofemoral Arthroplasty and Medial Patellofemoral Ligament Reconstruction for a Patient with Patellar Dislocation Combined with Hip Dysplasia: A Case Report of a Successful Outcome at 5-Year Follow-Up

**DOI:** 10.1155/2021/9970975

**Published:** 2021-09-03

**Authors:** Takuya Iseki, Tomoya Iseki, Shohei Okahisa, Shinichi Yoshiya, Shigeo Fukunishi, Toshiya Tachibana

**Affiliations:** ^1^Department of Orthopedic Surgery, Hyogo College of Medicine, Japan; ^2^Nishinomiya Kaisei Hospital, Japan

## Abstract

**Background:**

Posttraumatic patellar dislocation is rare, and consistent surgical strategy therefore has not been defined due to multifactorial factor. In this case study, we treated a case of a patellar dislocation with hip osteoarthritis and increased femoral anteversion by performing a two-staged surgery. In the first stage, total hip arthroplasty was performed, and in the second stage, simultaneous patellofemoral arthroplasty and medial patellofemoral ligament reconstruction using semitendinosus tendon autograft were performed. *Case Report*. A 56-year-old female patient who previously had right hip osteoarthritis complained of right knee pain after a fall. Radiographic examination showed lateral dislocation of the patella with osteoarthritic (OA) change in the patellofemoral joint and an excessive femoral anteversion with OA change on the right hip joint. Total hip arthroplasty was performed firstly to decrease femoral anteversion. Then, simultaneous patellofemoral arthroplasty and medial patellofemoral ligament reconstruction was performed for residual patellar dislocation and patellofemoral OA without tibiofemoral joint OA. At the time of the 5-year follow-up after surgery, the patient was able to walk with a wheelbarrow without any complications.

**Conclusion:**

To the best of our knowledge, this is the first case of a patellar dislocation with an increased femoral anteversion and patellofemoral OA treated by a combination of total hip arthroplasty, patellofemoral arthroplasty, and medial patellofemoral ligament reconstruction. The clinical outcome improved at 5 years after these surgeries. Therefore, these surgical options can be considered to be useful.

## 1. Introduction

Patellar dislocation is a rare condition and associated with multifactorial anatomical factors, and many surgical procedures for this condition have been reported [1, 2]. The increased femoral anteversion angle (FAA) has been reported as a risk factor of patellofemoral incongruity and patellar dislocation, because the internal torsion on the distal femur lateralizes the patellar joint and persistent force leads to increased pressure on the PF joint [3, 4]. Recently, there have been reports of patellar dislocation with PF osteoarthritis treated by total knee arthroplasty (TKA) [5–9]. However, it is controversial to perform TKA on a knee without tibiofemoral (TF) joint OA.

In this report, as a first stage of the procedure for posttraumatic patellar dislocation with increased FAA of the hip joint, total hip arthroplasty (THA) was performed to reduce the increased FAA. In the second stage of the procedure, the patellofemoral arthroplasty (PFA) and medial patellofemoral ligament (MPFL) reconstruction using semitendinosus tendon autograft were performed. At the 5-year follow-up after surgery, there was no complication including redislocation of patella or residual patellar instability.

## 2. The Case

A 56-year-old female who had been originally diagnosed with right hip OA was injured due to a fall and presented to previous emergency hospital. Patellar dislocation of the right knee was sustained, and closed reduction was performed under general anesthesia. However, the patella easily redislocated with flexion of the knee joint. She was transferred to our hospital three months after the accident, because persistent pain in the right knee still remained, and she had difficulty walking by herself with a wheelbarrow.

**Figure 1:**
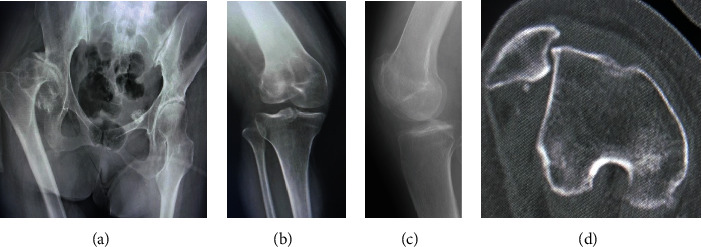
(a) Preoperative X-ray examination of the hip. (b) Preoperative X-ray examination of the right knee; anteroposterior view. (c) Preoperative X-ray examination of the right knee; lateral view. (d) Preoperative computed tomography of the right knee.

In terms of the range of motion of the right hip joint, contraction of the extension angle of -30°, abduction angle of -20°, and the external rotation angle of -30° were recognized (Table 1). The X-ray image of the right hip showed severe hip OA with Crowe type III dysplasia (Figure 1(a)). FAA of 44° was found on computed tomography (CT). Regarding the right knee joint, the patella was palpated on the lateral side of the femoral condyle. The range of motion on the right knee was -10° in extension to 80° in flexion with severe pain (Table 1). The X-ray and CT images of the right knee revealed lateral dislocation of the patella with OA change in the PF joint, while no apparent joint space narrowing was present in the TF joint (Figures 1(b)–1(d)). The assessment of patella instability on X-ray image such as the Insall-Salvati ratio and the congruence angle could not be measured due to dislocation of the patella. The preoperative patient reported outcome measurement; the 2011 Knee Society Score (2011 KSS) and the Modified Harris Hip score are shown in Table 1.

## 3. Surgical Strategy

**Figure 2:**
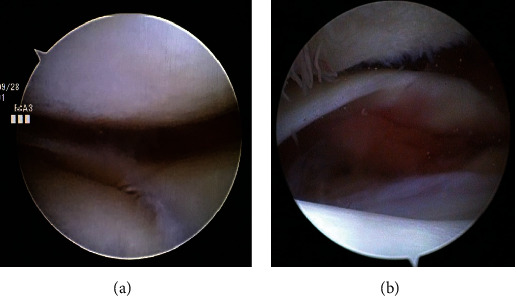
Arthroscopic view of the tibiofemoral joint: medial compartment (a) and lateral compartment (b).

**Figure 3:**
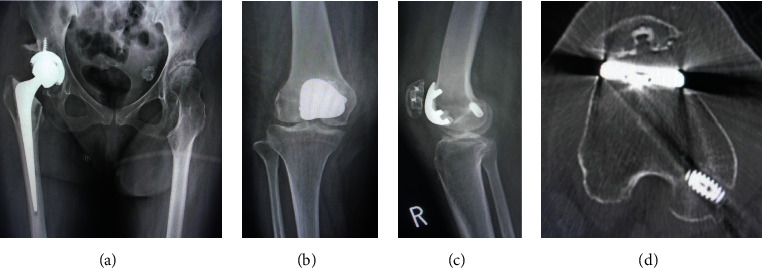
(a) X-ray of the hip after the first-stage surgery. (b) Postoperative X-ray of the right knee; anteroposterior view. (c) Postoperative X-ray of the right knee; lateral view. (d) Postoperative computed tomography of the right knee.

Regarding the surgical strategy addressing the multiple problems involved in the right limb, we planned a two-stage surgery. In the first stage, THA using a modified Hardinge approach (Stryker Orthopedics, Mahwah, NJ, USA) and subcutaneous adductor tenotomy were performed for the patellar dislocation caused by the hip contracture and the increased FAA. After the surgery, the FAA improved and normalized from 44° to 27°. The functional exercise of hip joint could be performed and attained the improvement of symptom at the hip joint in two months after the first surgery. However, the patella was still dislocated more than 30° when the knee was flexed. We performed the second-stage surgery. Firstly, an arthroscopic examination was performed to confirm the status of the TF and PF joints, showing ICRS classification grade 4 OA in the PF joint and ICRS classification grade 2 in the TF joint (Figures 2(a) and 2(b)). After arthroscopic examination, we performed PFA (Zimmer Gender Solutions ™ Patello-Femoral Joint, Warsaw, IN) with an onlay design using the midvastus approach. Anterior femoral bone cut was made perpendicular to Whiteside's line as the axis of the sagittal plane and the milling was performed using the dedicated guide. For patella replacement, patella bone cut was performed to reproduce the conventional patella thickness. Furthermore, despite adding the lateral release procedure through the inside of the capsule, the patella could not be reduced; thus, we decided to concomitantly perform a MPFL reconstruction. The MPFL was reconstructed using a double-strand semitendinosus tendon autograft harvested from the ipsilateral leg. The autograft was passed through the bone tunnel and fixed with an interference screw (Kurosaka profile, Depuy Synthes Mitek, Raynham, MA) on the femoral side. Two sockets for anchor insertion were prepared at the proximal and center portions of the medial aspect of the patella using a 2.9 mm diameter pin attached to the anchor system. These combined procedures successfully reduced the patella, and range of motion of the knee was from -5° to 95°. In the postoperative CT assessment, the patella tilting angle improved from 42.5° to 13.4° (Figures 3(a)–3(d)). At 5 years after surgery, no recurrence of patellar dislocation was observed, and the patient could walk with a wheelbarrow. Furthermore, there were improvements in the patient reported outcome measurement (Table 1).

## 4. Discussion

Patellar dislocation is a rare condition and associated with multifactorial anatomical factors such as valgus deformity of the knee, increased FAA, patella alta, ligament laxity, contracture of the lateral patellar soft tissues, hypoplasia of the lateral femoral condyle, and a laterally located tibial tubercle [1, 2]. Although procedures such as MPFL reconstruction and medial transfer of the tibial tubercle have been performed to attain reduction of the dislocated patella, surgical results have only been sporadically reported, and the optimal surgical strategy has not been defined.

Although the FAA in the normal population is between 14° and 16° [1, 2], it has been reported that patients with recurrent patellar dislocation had significantly larger FAA [10, 11]. Franciozi et al. reported that a FAA > 30° was a risk factor for inferior patient reported outcome in patients with recurrent patellar dislocation [12]. The mechanical basis of the increased risk likely lies with an increase in the *Q* angle resulting from the femoral anteversion, which exerts laterally directed forces on the patella. For this reason, in recent years, there have been some reports of femoral derotation osteotomy in addition to MPFL reconstruction as a treatment for recurrent patellar dislocation to decrease femoral anteversion [13]. In the present case, the increased FAA was corrected from 44° to 27° by THA combined with subcutaneous adductor tenotomy.

Some studies reported successful clinical cases of patellar dislocation treated by TKA with vastus medialis advancement [6–9]. Matsushita et al. reported satisfactory outcomes of TKA combined with MPFL reconstruction for patients with chronic patellar dislocation with valgus deformity of the knee [14]. However, there has been no report that performed simultaneous PFA and MPFL reconstruction for permanent patellar dislocation combined with PF joint OA. In the present case, radiological and arthroscopic findings showed a severe OA change in the PF joint, while little OA change was present in the FT joint. Additionally, there was no valgus deformity; therefore, PFA was performed with the FT joint left alone.

In recent reports, a new design incorporates the ability to externally rotate and translate the position of the femoral component, thus reducing the load on the lateral facet. In this design, a broad and relatively unconstrained trochlea is placed in the appropriate position so that the patella component can be stabilized within the groove. Although the present case had an anatomical problem of trochlear dysplasia as a factor inducing patellar instability, the PFA was able to effectively improve patello-femoral stability caused by the insufficient bony restraint.

In this case, although the TT-TG was measured as 28 mm, indicating tibial tubercle medializing osteotomy, patellar tracking and instability were not shown after the secondary surgery. Therefore, the tibial tubercle medializing osteotomy was not performed.

## 5. Conclusion

To the best of our knowledge, this is the first case of a patellar dislocation with increased femoral anteversion and patellofemoral OA treated by a combination of total hip arthroplasty, patellofemoral arthroplasty, and medial patellofemoral ligament reconstruction. The clinical outcome improved at 5 years after these surgeries. Therefore, these surgical options can be considered to be useful.

## Figures and Tables

**Table 1:** Pre- and postoperative range of motion and clinical score of the knee and hip joint.

	Preoperative	Postoperative
Right hip		
Flexion angle (°)	80	90
Extension angle (°)	-30	0
Abduction angle (°)	-20	20
External rotation angle (°)	-30	40
Right knee		
Extension angle (°)	-10	-5
Flexion angle (°)	80	95
Modified Harris hip score		
Total	16.5	60.5
Pain	10	40
Function	0	7
Activities	5	8
2011 knee society score		
Function	1	18
Satisfaction	6	26
Expectation	13	13
Activity	14	48
